# Lipoaspiration and Controlled Compressive Therapy in Lymphedema of the Upper Extremity: A Comprehensive Systematic Review

**DOI:** 10.7759/cureus.5787

**Published:** 2019-09-27

**Authors:** Antonio J Forte, Maria T Huayllani, Daniel Boczar, Gabriela Cinotto, Sarah A McLaughlin

**Affiliations:** 1 Plastic Surgery, Mayo Clinic Florida - Robert D. and Patricia E. Kern Center for the Science of Health Care Delivery, Jacksonville, USA; 2 Surgery, Mayo Clinic Florida - Robert D. and Patricia E. Kern Center for the Science of Health Care Delivery, Jacksonville, USA

**Keywords:** lipoaspiration, upper extremity, lymphedema, surgery, plastic surgery

## Abstract

Lipoaspiration followed by controlled compression therapy has been used to treat lymphedema of the upper extremity. We aimed to describe the studies reporting on outcomes of this procedure, in addition to reporting the differences with patients that were treated only with compressive therapy. The PubMed database was queried for studies that evaluated the use of lipoaspiration for upper extremity lymphedema. The keywords “aspiration lipectomy” AND “lymphedema” and synonyms in different combinations were used for the search. From a total of 129 articles, 13 met inclusion criteria. Ten studies reported outcomes of patients treated with lipoaspiration followed by compressive therapy, and three studies compared this procedure with patients that had only compressive therapy. A complete reduction of the edema in the affected limb was found in all the studies. Better results were found in patients who had undergone both procedures. This systematic review suggests that lipoaspiration is recommended for patients with upper extremity lymphedema of any cause in stage two after a long period of compressive therapy that did not produce additional edema reduction.

## Introduction and background

Lymphedema affects approximately three million people in the United States and 90 million people worldwide [1-2]. The cause of this chronic condition may be related to an embryologic abnormality of the lymphatic vessels (primary) or as a consequence of surgical treatment, lymph node resection, or radiotherapy for patients with cancer (secondary). This complication may impact the morbidity of patients [3]. Symptoms like heaviness, weakness, pain, sensory deficit of the extremities, anxiety, psychological compromise, maladjustment, and social isolation negatively impact the quality of life of patients diagnosed with lymphedema [2]. Moreover, the economic burden generated by lymphedema treatment is a considerable issue that may impact efforts to find the best therapy for this condition. Traditional surgical procedures have been limited to resection of skin and interstitium affected by lymphedema, although this procedure involves disfigurement and other complications due to the use of skin grafts that cover the resected areas [4]. Other recently used procedures, like microsurgical reconstructive techniques and vascularized lymph node transfers, are better suited for the early stages lymphedema, although they are more labor-intensive, time-consuming, expensive, and carry more risks after surgery [5]. Among all surgical options for lymphedema, power-assisted lipoaspiration was proposed, with the intention of removing adipose tissue from chronic lymphedema [6]. 

The purpose of this study is to systematically review and summarize the studies to date that report outcomes regarding surgical treatment with lipoaspiration and controlled compressive therapy, and to describe the indications to perform this procedure in patients with lymphedema of the upper extremity. In addition, we describe the surgical technique, controlled compression therapy, and volume measurement techniques used on the reported studies.

## Review

Materials and Methods

Study Selection

This systematic review included all studies evaluating the efficacy of lipoaspiration surgery in patients with upper extremity lymphedema following the Preferred Reporting Items for Systematic Reviews and Meta-Analyses (PRISMA) guidelines for article identification and final selection. Studies were included if they reported preoperative and postoperative volume measurements of the limb in patients who underwent lipoaspiration to treat upper extremity lymphedema. All studies were written in English. Studies were excluded if they reported results that were not specific for upper extremity lymphedema, if lipoaspiration was used in combination of any other surgical treatment, or if they were reviews.

Data Sources and Search Strategy

A comprehensive systematic review was conducted by one author (Maria Teresa Huayllani) on July 23, 2019, in the PubMed database searching for articles reporting on lipoaspiration for treatment of upper extremity lymphedema. A search strategy was generated using the following terms: (Lipectomies[Title/Abstract]) OR Aspiration Lipectomy[Title/Abstract]) OR Aspiration Lipectomies[Title/Abstract]) OR Lipectomies, Aspiration[Title/Abstract]) OR Lipectomy, Aspiration[Title/Abstract]) OR Aspiration Lipolysis[Title/Abstract]) OR Lipolysis, Aspiration[Title/Abstract]) OR Suction Lipectomy[Title/Abstract]) OR Lipectomies, Suction[Title/Abstract]) OR Lipectomy, Suction[Title/Abstract]) OR Suction Lipectomies[Title/Abstract]) OR Lipolysis, Suction[Title/Abstract]) OR Suction Lipolysis[Title/Abstract]) OR Liposuction[Title/Abstract]) OR Liposuctions[Title/Abstract]) OR Lipoplasty[Title/Abstract]) OR Lipoplasties[Title/Abstract]) AND ((lymphedema[Title/Abstract]) OR lymphoedema[Title/Abstract]). Identified studies were uploaded into EndNote (Clarivate). Two independent reviewers (Maria Teresa Huayllani, Daniel Boczar) selected the final studies. Manuscripts were screened manually by one author (Maria Teresa Huayllani) and selected according to inclusion and exclusion criteria in a two-step process. First, studies were reviewed based on the title and abstract. Second, the full text of the selected studies was screened for the final selection. A second author (Daniel Boczar) reviewed articles that were questionable to include and, according to selection criteria, both reviewers came to an agreement for the final decision. 

Data Pooling and Data Analysis

Relevant data were extracted and pooled. The variables selected to describe studies to date included author, year of publication, type of study, number of patients, age of patients, cause of disease, stage of lymphedema, duration of lymphedema, lipoaspirate volume, measurement tool, follow-up, and outcomes.

Results

**Figure 1:**
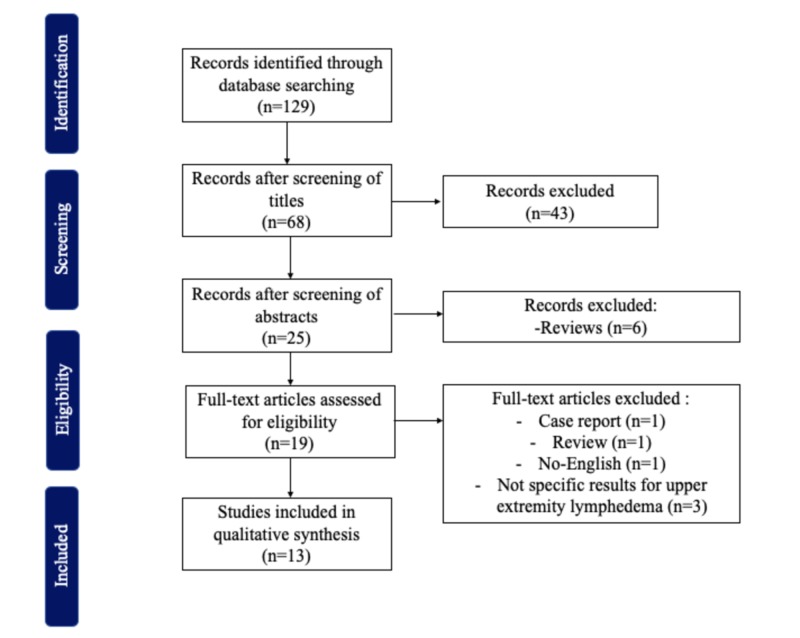
Inclusion and exclusion criteria

We found 129 articles in our PubMed search, 13 of which met inclusion criteria (Figure 1). All included studies were published between 1997 and 2018. Study descriptions are provided in Tables 1 and 2. In 10 studies, patients with lymphedema were treated with lipoaspiration first, followed by controlled compressive therapy (Table 1) [7-16]. The other three studies included comparisons between outcomes of patients who underwent lipoaspiration with controlled compressive therapy and patients treated with controlled compressive therapy alone (Table 2) [17-19]. Most studies evaluated outcomes in patients with lymphedema after breast cancer treatment, with the exception of two studies that included one patient with congenital lymphedema and one with lymphedema secondary to thyroidectomy [11-12]. Most of the patients with lymphedema were on stage two or three, with a mean duration of lymphedema ranging from 7 to 11 years. Not all studies reported the mean volume of lipoaspiration, but when reported, it ranged between 1,131 and 2,250 mL [8-9, 12, 14-16]. Nine studies compared preoperative and postoperative volumes of affected arms using the water displacement technique, three studies used the 4 cm truncated cone circumferential method and one used plethysmography [7-19]. Moreover, changes in the bioimpedance, quality of life, incidence of infection, range of motion, and skin blood flow were also evaluated in some studies [7, 9-11, 15-17]. Overall, there was a complete reduction of edema in the affected limb, reaching to more than 100% of relative reduction in all patients who underwent lipoaspiration followed by controlled compression therapy. In two studies, results also found a decrease in the scores of bioimpedance (L-Dex) [7, 11]. Interestingly, an increase in quality of life, a decrease in incidence of infection, an improvement in range of motion, and an increase in skin blood flow were found in patients treated with lipoaspiration followed by controlled compressive therapy [9-10, 16-17].

**Table 1:** Studies to date reporting liposuction with controlled compressive therapy outcomes R, retrospective; P, prospective; NS, not specified; LS, liposuction; CCT, controlled compressive therapy; ISL, International Society of Lymphology; VAS, Visual Analog Scale; ADL, activities of daily living; NHP, Nottingham Health Profile; PGWB, Psychological General Well-Being; HAD, Hospital Anxiety Depression Scale.

Author	Year	Type of study	Number of patients	Age	Cause	ISL Stage	Duration of lymphedema (mean in years)	Lipoaspirate volume (mean in ml)	Measurement Tool	Follow-up (months)	Outcomes
Sen Y et al. [7]	2018	R	5	Mean: 64	Breast cancer	2, 3	8.5	NS	4 cm truncated cone circumferential measurements, bioimpedance	NS	Volume decreased, L-Dex measures decreased
Hoffner M et al. [8]	2018	P	105	Mean: 64	Breast cancer	2	10	1,831	Plethysmography	60	Volume decreased
Hoffner M et al. [9]	2017	P	60	Mean: 64	Breast cancer	2	10	1,362	Water displacement technique, 36-item short form health survey (SF-36)	12	Volume decreased, better quality of life
Lee D et al. [10]	2016	P	130	Mean: 63	Breast cancer	NS	8.8	NS	Water displacement technique	6	Volume decreased, incidence of erysipelas decreased
Boyages J et al. [11]	2015	P	15	Mean: 57.8	Breast cancer (n=14), congenital (n=1)	2, 3	9.1	NS	4 cm truncated cone circumferential measurement, bioimpedance	18	Volume decreased, L-Dex measures decreased
Schaverien MV et al. [12]	2012	P	12	Mean: 57	Breast cancer (n=11), thyroidectomy (n=1)	NS	7	1,131	4 cm truncated cone circumferential measurements	60	Volume decreased
Damstra RJ et al. [13]	2009	P	37	Mean: 59	Breast cancer	2	8.2	NS	Water displacement technique	12	Volume decreased
Bagheri S et al. [14]	2005	P	20	Mean: 50	Breast cancer	NS	11	1,724	Water displacement technique	12	Volume decreased
Brorson H et al. [15]	1997	P	28	Mean: 63	Breast Cancer	NS	NS	2,250	Water displacement technique	12	Volume decreased
Brorson H et al. [16]	1997	P	12	Median: 62	Breast cancer	NS	8	2,060	Arm volumes: water displacement technique. Laser Doppler imaging (LDI) for skin blood flow assessment	12	Volume decreased, skin blood flow increased, incidence of cellulitis decreased

**Table 2:** Studies to date reporting outcomes of liposuction with controlled compressive therapy versus controlled compressive therapy alone R, retrospective; P, prospective; NS, not specified; LS, liposuction; CCT, controlled compressive therapy;  ISL, International Society of Lymphology; VAS, Visual Analog Scale; ADL, activities of daily living; NHP, Nottingham Health Profile; PGWB, Psychological General Well-Being; HAD, Hospital Anxiety Depression Scale.

Author	Year	Type of study	Number of patients	Age	Cause	ISL Stage	Duration of lymphedema (mean in years)	Lipoaspirate volume (mean in ml)	Measurement Tool	Follow-up (months)	Outcomes
Brorson H et al. [17]	2006	P	49 (LS+ CCT: 35, CCT: 14)	Mean, LS+CCT: 65, CCT: 66	Breast cancer	2	LS+CCT: 7.4, CCT: 7.9	NS	Water displacement technique, goniometer for range of motion, VAS, ADL, NHP, PGWB, and HAD for quality of life	12	Volume decreased, better range of motion, better quality of life, less anxiety.
Brorson H et al. [18]	1998	P	20 (LS+CCT: 11, CCT: 9)	Mean, LS+CCT: 61, CCT: 64	Breast cancer	2	LS+CCT:7.5, CCT: 7.1	NS	Water displacement technique	18	Volume decreased
Brorson H et al. [19]	1998	P	28 (LS+CCT: 14, CCT: 14)	Mean, LS+CCT: 54, CCT: 56	Breast cancer	2	LS+CCT: 7.8, CCT: 7.9	NS	Water displacement technique	18	Volume decreased

Surgical Technique

The first step prior to performing a lipoaspiration should be general anesthesia after limb exsanguination [11]. To minimize blood loss, the “dry technique”, or the tourniquet in combination with the tumescent technique, which involves a ratio infiltration volume to aspirate volume of 3:1 through the infiltration of 1 L of Ringer's lactate with 1 ml of adrenaline 1:1,000 and 300 to 500 mg of lidocaine, can be applied [8, 13, 20]. The power-assisted lipoaspiration should start from the distal part of the arm, covering the entire arm circumferentially [16]. Blunt cannulas with an outer diameter of 3 mm to 4 mm or 4 mm to 5 mm, two to three openings distally and with a length of 15 and 25 cm can be used [8, 11]. Thirty to forty stab incisions of approximately 3 mm on the affected arm following the Gaussian distributions are usually performed to remove subcutaneous tissue while connected to a vacuum pump [12]. Preoperative measurements will determine the lipoaspirate volume needed to equalize the affected with the unaffected limb. Antibiotics, like penicillin derivatives or cephalosporin, should be given in the first 24 hours and then, taken orally for 10 to 14 days after surgery until the excisions are healed [8, 15]. 

Controlled Compression Therapy

Compression therapy is important because of the need to maintain results after lipoaspiration. During follow-up, compression garments should be used to continue to prevent recurrence of lymphedema. Immediately after surgery, compression garments of 30 mm Hg to 40 mm Hg (class two) should be applied to the affected arm prior to tourniquet release [9]. Measurement of these garments is usually made in the nonaffected arm 2 weeks before surgery, and then at 3, 6, 9, and 12 months after surgery until complete reduction is achieved [8, 20]. All studies in this systematic review had more favorable results with the use of controlled compression therapy after lipoaspiration.

Volume Measurements

The most common technique used for measuring upper extremity volume is the water displacement technique, which is defined as the procedure that measures the volume of water displaced after immersion of the whole arm into a water-filled container [8]. The arm is submerged until the fingertips reach the bottom of the container; the displaced water is then collected and weighed on a scale to the nearest 5 g, corresponding to 5 ml [9]. The second most common upper extremity volume measurement technique is called the 4 cm truncated cone circumferential measurement. This technique originates from the visualization of the arm as a truncated cone, where the circumference of the proximal and distal limb limits the cone and an established equation determines the final volume of the arm [21]. Plethysmography is a validated method of measuring the volume of the limb in patients with lymphedema, though it was not commonly used to measure volume in these studies [8]. Finally, bioimpedance spectroscopy (L-Dex) is a technique that has been successfully integrated in the assessment of breast cancer survivors with lymphedema [22]. This technique calculates extracellular fluid in the extremity using a low-voltage electrical current. The L-Dex measurements are the impedance ratio comparing the unaffected with the affected extremity, with the unaffected extremity acting as a control [7, 11].

Discussion

The purpose of this review is to evaluate whether treatment of upper-extremity lymphedema with lipoaspiration is efficient. Studies have found a complete reduction of edema in the affected limb of patients with chronic upper extremity lymphedema, reducing the arm volume a little more than nonaffected arms.

Patients who were evaluated after lipoaspiration followed by controlled compressive therapy were at stage two or three of the disease and had a mean lymphedema duration ranging from 7 to 11 years. These findings support the idea that lipoaspiration is better suited in late stages of disease and as a second treatment option, when the treatment with controlled compressive therapy alone did not work initially. This statement is based on lymphedema pathophysiology suggesting that the absence of axillary lymph nodes after excision and radiotherapy result in a slower flow rate of blood and lymph, thus promoting lipogenesis and deposition of fat due to transformation of macrophages [23]. As a result, after an early stage of extravasation of fluid rich in protein, adipose tissue hypertrophies, generating a lymphedema with absent or less pronounced pitting (stage two) that over time will activate inflammatory cells and fibrocytes increasing the fibrosis and connective tissue in the affected arm of chronic patients (stage three) [24]. 

Regarding the volume of lipoaspiration, the excess preoperative volume found in each affected arm, compared with the nonaffected arm, helped guide the exact fat volume to aspirate [8-9, 12, 14-16]. On the other hand, an inverse correlation between excess volume reduction 5 years after surgery and preoperative excess volume was found previously [8]. This means that reestablishment of the previous volume is better achieved when lymphedema is less pronounced. However, complete reduction of excess volume was found even with high volumes of lipoaspiration [15-16]. 

Two studies used bioimpedance to compare pre- and postoperative outcomes. Sen et al. revealed a decrease of L-Dex in almost all patients, with the exception of one, who reported a median change before and after surgery of -10.4% (range, +52.2 to -60.5%) [7]. Similar to this, Boyages et al. reported a decrease in L-Dex measures at 12 months postsurgery, after an initial increase at four weeks postsurgery, though these comparisons were not statistically significant [11]. The first study showed a wide range of L-Dex results and the second showed a decrease of measurements at a longer period of time after surgery. A possible explanation for these inconsistent results may be the presence of fat, rather than water in the interstitial space, which makes bioimpedance an unproductive tool to assess patients with stage two or three lymphedema of the upper extremity. 

The most notable finding reported in this systematic review is the impact of lipoaspiration on quality of life. We found two studies that reported a better quality of life in patients with lipoaspiration and controlled compression therapy. Hoffner et al. utilized the short form health (SF-36) questionnaire to assess patient quality of life before and after surgery [9]. They reported an improvement in scores for physical functioning, pain, vitality, social functioning, and mental health after 12 months of lipoaspiration. Moreover, in 2006, Brorson et al. described a significant correlation between reduced edema volume and improved quality of life parameters such as pain, range of motion, and perceived health problems [17]. Furthermore, a significant decrease in anxiety was detected after lipoaspiration and compression therapy. These results differ from those of Sitzia and Sobrido, who did not find a significant association between edema volume reduction and quality of life [25]. One possible cause of this difference between studies may be the fact that the sample of patients evaluated in the last study were those treated with conservative treatment in comparison to the others, suggesting that lipoaspiration in combination with compressive treatment may have a better impact on the quality of life.

A decrease in the incidence of erysipelas and cellulitis after lipoaspiration and compression therapy was found in two studies [10, 15]. A possible explanation for this finding is the increase of skin blood flow present in lymphedema patients, which brings immune cells to potential future infection sites caused by accumulation of fluid, fat, and inflammatory cells [16, 26]. In addition, the use of compression garments may contribute to a lower likelihood of skin and subcutaneous infection, as that could benefit lymph fluid dynamics, avoiding the lymphatic stasis in the affected extremity. 

When analyzing outcomes, all studies found a potential benefit in volume reduction to treat patients with chronic lymphedema. Hoffner et al. reported a rapid decline of preoperative mean excess volume, from 1,373±73 mL to 75±35 mL 1 month after surgery, to a complete volume reduction of -26±40 mL at 3 months [9]. In this study, 49 (82%) patients reduced excess volume completely. In contrast, in a recent study that followed patients until 5 years after surgery, a high mean excess volume reduction, from 1,573±645 mL to -188±300 mL, was reported, which corresponds to a 117%±26% reduction 5 years after surgery [8]. This suggests that lipoaspiration may also be beneficial in the long term if patients follow the treatment protocol and continue controlled compressive therapy.

Brorson et al. identified the difference between outcomes after lipoaspiration and controlled compressive therapy versus controlled compressive therapy only in three studies [17-19]. They found a statistical difference in volume reduction between these two groups at 12-month follow-up. The postoperative relative reduction for patients with lipoaspiration and controlled compressive therapy were 103%, 115%, and 113%, respectively, in the three studies, whereas in the group treated with controlled compressive therapy only were 50%, 54%, and 47%, respectively (P<.005) [17-19].

Regarding complications, no major surgical complications were reported in any case. Complications such as transient paresthesia in the operated arm, temporary superficial abrasions in the wrist caused by the compression garment, and postoperative pneumonia and dyspnea were reported in few cases [15]. Blood transfusion was needed for eight patients whose volume of lipoaspiration exceeded 2,000 mL, probably due to the lack of tourniquet during the procedure [15]. The application of tourniquet has demonstrated to reduce completely the need for blood transfusion [8]. On the other hand, Brorson et al. reported a patient who had two local subcutaneous glandular metastases excised from the adipose tissue in the upper arm and later developed generalized metastatic disease [16].

Strengths and Limitations

This comprehensive systematic review reported all English-language manuscripts, to date, assessing the efficacy of lipoaspiration as a treatment option for upper extremity lymphedema. Limitations of our study include the heterogeneity between studies regarding the measurement tool used, the follow-up of results, and the protocol established in each study that made statistical analysis difficult to compare results between studies. In addition, there are inherent limitations in review methodology because of search, selection, and publication biases. However, we believe this reported data is valuable, as it describes the primary findings and benefits of lipoaspiration.

## Conclusions

Lipoaspiration treatment followed by controlled compressive therapy should be recommended in stage two of chronic lymphedema as a second option after conservative therapy that demonstrates no more benefit in volume excess reduction. Benefits of complete volume excess reduction start 3 months after surgical treatment and remain as long as controlled compressive therapy is continued. Other advantages related to this procedure are the improvement in the quality of life, range of motion, and skin blood flow, as well as the decrease in incidence of infection.
